# ProteinCT: An implementation of the protein circuit topology framework

**DOI:** 10.1016/j.mex.2022.101861

**Published:** 2022-09-16

**Authors:** Duane Moes, Elnaz Banijamali, Vahid Sheikhhassani, Barbara Scalvini, Jaie Woodard, Alireza Mashaghi

**Affiliations:** a Medical Systems Biophysics and Bioengineering, Leiden Academic Centre for Drug Research, Faculty of Science, Leiden University, Leiden 2333 CC, the Netherlands; b Department of Medical Biochemistry and Biophysics, Karolinska Institutet, Stockholm 17177, Sweden

**Keywords:** Structural biology, Topology, Circuit topology, Protein, Contact

## Abstract

The ability to describe the topology of a folded protein conformation is critically important for functional analysis, protein engineering, and drug design. Circuit topology is a unique topological framework which is widely applicable to protein analysis, yet a state-of-the art implementation of this concept is lacking. Here, we present an open-source Python-implemented circuit topology tool called ProteinCT. The platform provides a method for acquiring, visualizing, analyzing, and quantifying circuit topology data from proteins of interest. We mapped the universe of human proteins to a circuit topology space using conventional hardware within a few hours, demonstrating the performance of ProteinCT. In brief,

• A Python-implemented circuit topology tool is developed to extract global and local topological information from a protein structure file.
• Modules are developed to combine topological information with geometric and energetic information.
• It is demonstrated that the method can be efficiently applied to a large set of proteins, opening a wide range of possibilities for structural proteomics research.

## Graphical abstract

**Figure:**
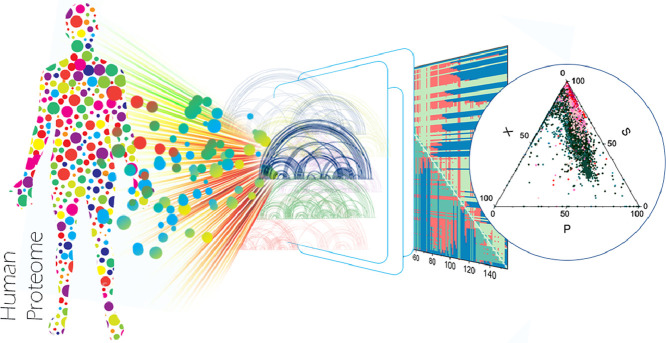

Specifications table

**Table:** 

Subject Area:	Bioinformatics
More specific subject area:	*Structural Bioinformatics*
Method name:	*Circuit Topology of Proteins*
Name and reference of original method:	*Mashaghi, A., Van Wijk, R.J. and Tans, S.J. (2014) ‘Circuit topology of proteins and nucleic acids’, Structure, 22(9), pp. 1227–1237. doi:10.1016/j.str.2014.06.015.* *Scalvini, B., Sheikhhassani, V. and Mashaghi, A. (2021) ‘Topological principles of protein folding’, Physical chemistry chemical physics: PCCP, 23(37), pp. 21316–21328. doi:10.1039/d1cp03390e.*
Resource availability:	*Source code is freely available at*https://github.com/circuittopology/circuit_topology

## Method details

### Background

Understanding the diversity in biomolecular structures and its functional consequences is one of the most pressing scientific challenges in biology today [1], [2], [3]. Translating this complexity into a functional and accurate topological framework allows us to understand the rich networks of structural relationships and provides insights into folding mechanisms, conformational dynamics, and folding stability, ultimately facilitating protein and drug design [4]. The (mis)folding of a protein is a phenomenon that is generally not well understood, with many elusive influencing factors. Since misfolding of proteins is at the core of many cancers and neurodegenerative diseases [5], pharmaceutical research would certainly benefit from further insights into protein folding mechanisms, facilitated by topological discoveries.

**Fig. 1:**
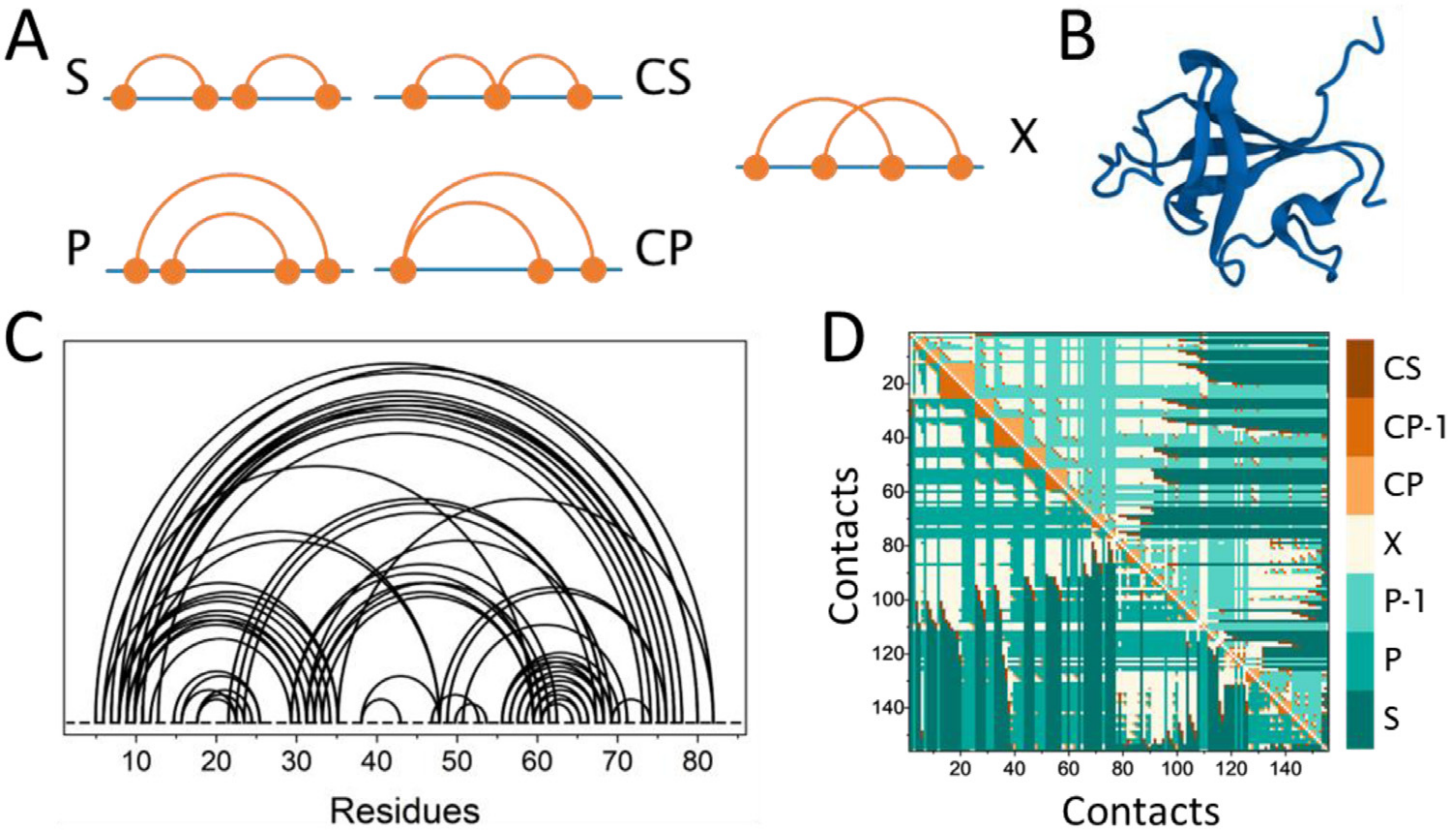
Human proteome mapped to a circuit topology space. (A) Different possible configurations of a pair of contacts in CT map. Three main configuration are Series (S), Parallel (P), and Cross (X). (B) Cartoon representation of the bovine phosphotransferase (PBD code: 1PNJ). (C, D) Circuit diagram and CT matrix calculated for the PDB entry 1PNJ.

A newly devised topological framework called Circuit Topology (CT) [6], [7], offers a wealth of information on protein structures that other topological theories such as knot theory do not provide [8]. It defines specific types of pairwise relations between intramolecular contacts, in which there are three basic variants: Parallel (P), Series (S), Cross (X), and two additional related ones, Concerted Parallel (CP) and Concerted Series (CS) (Fig. 1A). Any pair of intramolecular contacts can be defined as one of these five relations, such that a transition between these variations would involve the formations and/or rupture of fixed contacts and/or of the protein backbone. Recent analyses have shown that various structural benchmarks of proteins such as contact order, size and CT parameters can be used as (un)folding rate predictors [9], [10], [11], [12], [13], [14]. While contact order depends on the sequence distance between residues, CT is independent, but can be combined with other geometric and energetic measures and sequence information. CT thus confers an inherent advantage in estimating folding rates and number of unfolding paths of a folded linear macromolecule [12], [15], [16]. Finally, recent evidence shows that circuit topology can predict pathogenicity of missense mutations, which often alter the folding stability of a protein, in addition to other properties such as aggregation propensity, binding affinity to other proteins and/or ligands, catalysis, and dynamic aspects relevant to protein function [17].

### ProteinCT toolbox

No available tool allows an implementation of circuit topology calculation that provides both the classical intramolecular contact maps and circuit topology-based information in a simple format that can be used in further biological research without necessitating extensive prior knowledge. Here, we present an open-source Python-implemented circuit topology tool called ProteinCT. All algorithms under the ProteinCT tool are built using open-source Python libraries such as Scipy, Matplotlib and BioPython. The tool combines multiple principles of CT in an easy to use, customizable program that can be used by a non-specialist audience. The platform provides a method for acquiring, visualizing, analyzing, and quantifying CT data from proteins of interest. The algorithms can automatically process large protein structures efficiently and can be used for many purposes, including simulation analysis. ProteinCT is available for download at: https://github.com/circuittopology/circuit_topology

Compared to our previous implementation of protein CT [18], ProteinCT adds many novel functionalities, with the introduction of the CT analysis and easy-to-use features for plotting the related information. The ability to find and filter out long-range/short-range contacts, contacts within or between specific secondary structures using a STRIDE secondary structure map [19], and contacts exhibiting attractive vs. repulsive Thomas-Dill energies of interaction [15], in addition to finding CT circuits [8], are functions not seen before. More recently, a study has been done that was focused on expanding the circuit topology framework from single protein chains to macromolecular complexes and condensates [20]. This expansion in theory has also been adapted in the ProteinCT tool, which allows for macromolecular complexes and condensates be analyzed using the regular functions. While there are numerous ways to use the program, the steps below are necessary regardless of the use case.

### Input structures

ProteinCT uses PDB and macromolecular Crystallographic Information File (mmCIF) formatted files that can either be manually downloaded from the wwPDB [21], downloaded directly through a built-in function, or uploaded from locally stored files. Even though a PDB file is a valid input, it is still recommended that mmCIF files are used. PDB files were formatted in 1976 to fit the restriction set by computer punch cards to 80 characters per line. In 2014, the Protein Data Bank switched from PDB as a standard format and in 2019 they also stopped accepting any new depositions in the PDB format. Moreover, the PDB file format has not been modified to support any new content since November 21, 2012. Beside the discontinued support, the PDB format also experiences many problems and limitations regarding size of structures, number of chains/residues/atoms, complex chemistry, new experimental methods, and duplicate/missing atoms. The format that replaced PDB as the official file format is the macromolecular Crystallographic Information File (mmCIF), which experiences no difficulty with the number of structures and is continually being updated to include new content, ensuring backwards compatibility. Old users will also be advised to switch their current saved proteins over to the mmCIF format; however, some molecular modeling programs still only support the PDB format. Thus, inclusions have been made to the program to ensure backwards compatibility with the PDB format, such that the tool functions with both the mmCIF and the PDB format. One can quickly import the specified protein structure using the *retrieve_chain()*, function. When dealing with structures containing more than one chain, users can provide a chain identifier.

### Contact identification

ProteinCT outputs are produced in the form of arrays that contain the locations of intramolecular contacts. Intramolecular contacts are defined based on several methodologies; the default option defines contacts based on distance between atoms. Three separate cutoff distances are implemented: the maximum distance between two atoms that counts as an atom-atom contact (default value: 4.5 Å), the number of atom-atom contacts between two residues to count as a residue-residue contact (default value: 5), and the number of direct residue neighbors to exclude when determining contacts (default value: 3).

Other options include *centre of geometry*, where contacts are based on a maximum distance between geometrical centres of two respective amino acids; and *centre of mass*, which defines contacts based on distance between two amino acids and their respective weight distribution. Contacts are indexed by amino acid numbering described in the protein structure files.

### Filtering

After intramolecular contacts are defined, multiple filtering options are available that filter out certain biological or physical properties. These properties can be then used to examine the effects of certain changes or limitations on protein folding and the intramolecular dynamics. Properties that can be examined and filtered out include: Short-range and long-range contacts, using the function *length_filter(),* intramolecular contacts exhibiting attractive vs. repulsive Thomas-Dill energies of interaction using the function *energy_cmap()* and intramolecular contacts related to certain secondary structures using *secondary_struc_filter()*.

### Topological assignment

These intramolecular contacts are then used to determine CT relationship of two contacts; this is exported in matrix form, with each index containing one of five relation types, as illustrated in Fig. 1 (P/P^−1^, S, X, CP/CP^−1^, CS).

### Post processing

Optionally, the information can be automatically exported to CSV format. The CT matrix shows the CT relation type between contact *i* on the X-axis and contact *j* on the Y-axis. For example, in Fig. 1 the intramolecular contacts and their respective CT relations (Fig. 1C and 1D) are plotted for a bovine phosphotransferase ([22] [PDBcode: 1PNJ]) together with the 3D structure (Fig. 1B) generated by Pymol. The contact map defines formation of a circular line between two residues when a contact is found; the map shows which contacts are encapsulated and if there are any long-range contacts present.

### Proteome analysis

**Fig. 2:**
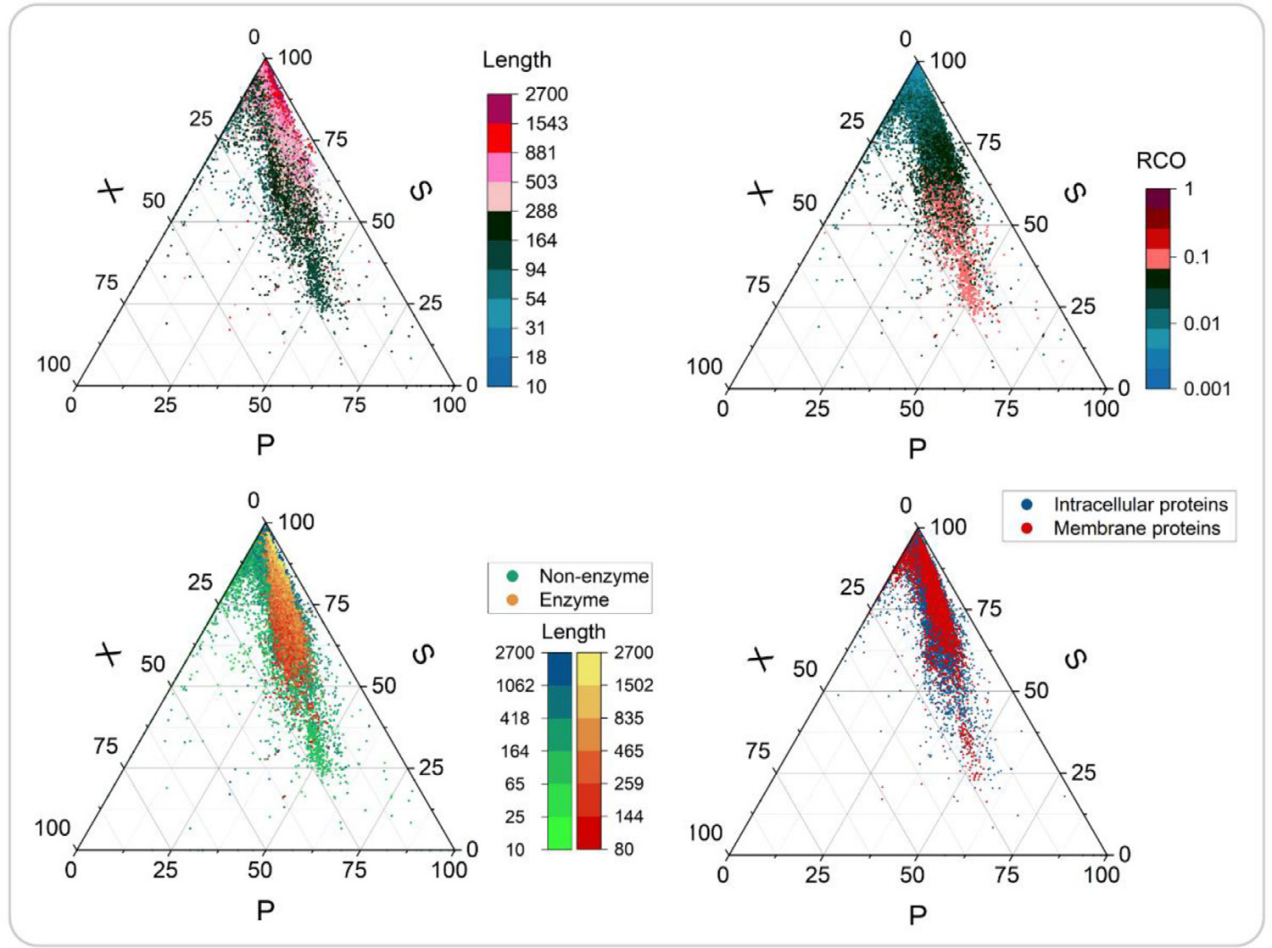
Ternary plots of PSX content of the entries from AlphaFold (n = 20500). Data points are color-mapped based on the Length, Relative Contact Order (RCO), function (enzyme or non-enzyme), and cellular location of the proteins. 2556 duplicate files were removed from the original dataset (n=23056).

To demonstrate the efficiency of our algorithm, we ran the code on all 23,056 entries of Alphafold [23] and mapped the universe of human proteins to a circuit topology space and performed a preliminary analysis of basic properties: the fraction of P, S, and X relations (Fig. 2). To get to this simplified, yet rich representation, we reduce CP to P and CS to S, a simplification which has been previously described for circuit topology. The run time for this data set (n = 23,056) on a 2021 Macbook Air was 4 h. Here, our aim is to demonstrate the efficiency of our implementation and thus we leave the detailed analysis of the topological makeup of the human proteome for future studies.

### Combining topology with other molecular properties

**Fig. 3:**
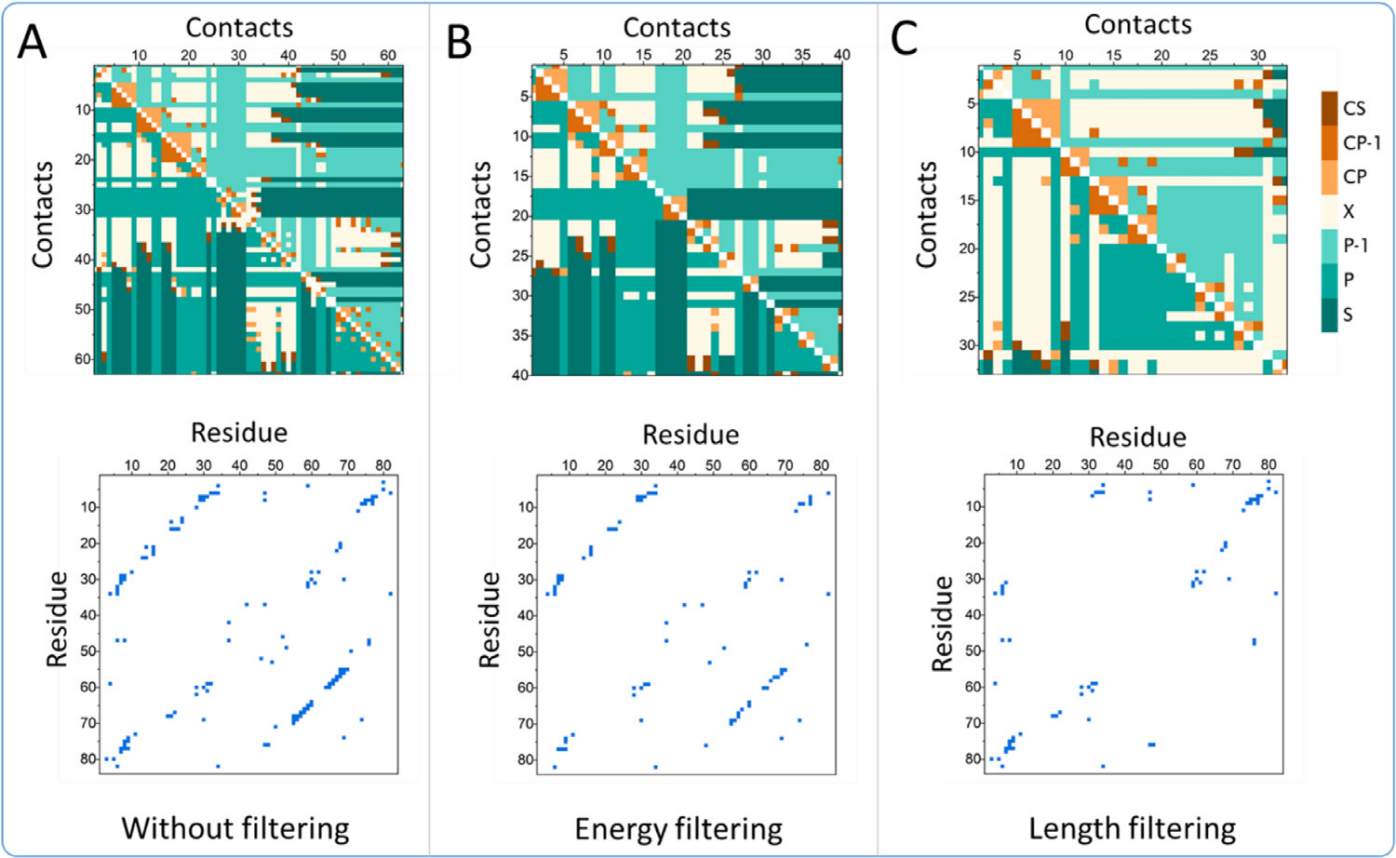
Circuit topology of the bovine phosphotransefare (PDB code: 1PNJ). (A) Contact matrix and circuit topology matrix before filtering. (B) Contact matrix and circuit topology matrix after filtering out repulsive Thomas-Dill energies. (C) Contact matrix and circuit topology matrix after filtering out short-range contacts (<25 amino acids).

Next, we provide an example of how various filers can be applied and examine the consequences on the circuit topology of a protein. We analyse the same protein example presented in Fig. 1, i.e., (PBD code: 1PNJ), keep in mind that we use the same protein throughout this article solely for consistency purposes, 1PNJ does not carry any significant preexisting importance. Fig. 3 shows the results after applying a filter that removes repulsive Thomas-Dill energy contacts [15], and after filtering out contacts that consist of two residues within a distance of 25 residues. One can quickly notice the effect both methods have on the number of residues in contact. The length filtering method (Fig. 3C) shows the biggest difference. The residue-residue contact matrix shows a large gap in the middle, that is caused by the exclusion of short-range contacts, but more importantly one can also see that the circuit topology matrix shows a relatively high number of cross contacts (X) when compared to the previous methods. Here we demonstrate three of these filtered used in parallel, but the reach of possibilities is far wider that currently shown. If needed users are able to use the desired filter types in a desired order and chain them together in order to attain the demanded level of analysis.

### Local circuit topology analysis

**Fig. 4:**
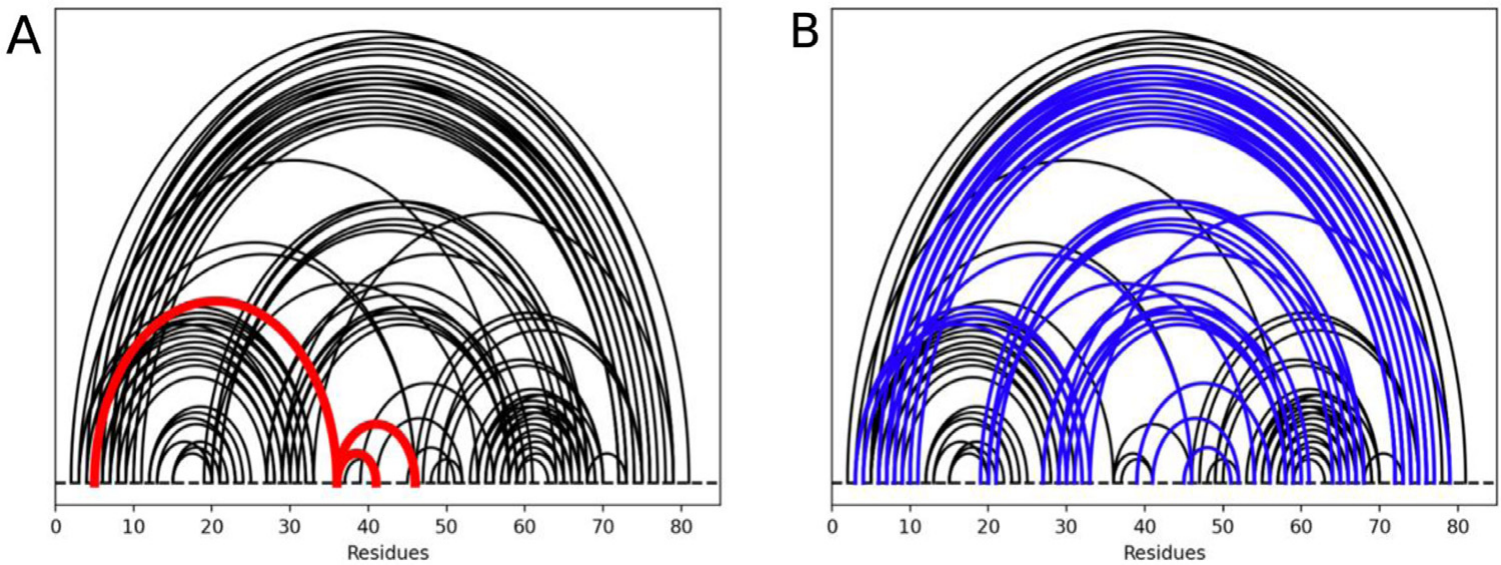
Local circuit topology of the bovine phosphotransferate (PDB code: 1PNJ). (A) Contact map with residue contacts found that either end or begin in residue #36 highlighted in red. (B) Contact map showing blue curves that are in cross relation with at least one red curve in (A).

One of the newer functions added to the ProteinCT environment was the ability to perform circuit topology analysis on a more local level present within existing residue circuit topology. Local circuit topology can be used to seek out any differences between mutated variants of certain residues and their respective contacts within proteins. It is speculated that differences in local topology of various mutants of the same protein can reflect the nature of polymer folding in the biological environment [17]. To demonstrate this functionality within the ProteinCT tool, an example analysis was done using the bovine phospotransferase (PDB code: 1PNJ). Fig. 4 plots the local cross circuit topology of residue 36; contacts shown in blue are in cross with any of the contacts for which residue 36 is one of the two sites. One can quickly notice the significant number of cross contacts related to only 1 residue site within the protein, a significant chunk of all contacts within the protein system can be seen as cross contacts related to the ones stemming from residue 36. A large number of contacts in cross was seen to be indicative on average of pathogenicity upon mutation [17].

## Conclusion

We provided an efficient implementation of protein circuit topology and described how it can be applied to a wide range of proteins. The topological data extracted in this way can be subjected to subsequent statistical analysis and machine learning procedures. The simplest analysis includes counting the number of contact pairs that take a given topological relation. Higher order analysis are also possible including circuit decomposition [8], [14] and finding patterns in the topology matrix. The identified topologies may also be subjected to comparative analysis. Various distance measures have been developed which can be used for comparing two topologies in different contexts. [8], [24], [25] Furthermore, topological information can be merged with geometric, energetic and chemical information to gain insights into biology. Finally, topological changes can be followed over time for analysis (un)folding and protein dynamics [26].

In conclusion, ProteinCT provides a user-friendly tool for extracting topological information from a given protein structure; the topological information can then be correlated to physical and functional properties of proteins. One can also combine topological studies with mutation analysis to gain insights into disease processes. Mutations may affect topology by stabilizing or destabilizing contacts; topology can in turn be used to predict whether mutation in a residue would destabilize a protein and thus may be pathogenic. Furthermore, ProteinCT provides a single tool that allows for traditional analysis and visualization of intra- and intermolecular contacts and interfaces of biomolecular complexes, combined with novel forms of analysis provided by the CT framework. It can be applied very easily and efficiently to both experimental and simulated 3D protein structures.

## Declaration of Competing Interest

The authors declare that they have no known competing financial interests or personal relationships that could have appeared to influence the work reported in this paper.

## Data Availability

Data will be made available on request.
